# Cytotoxic Effects of Hellebrigenin and Arenobufagin Against Human Breast Cancer Cells

**DOI:** 10.3389/fonc.2021.711220

**Published:** 2021-08-26

**Authors:** Yu Zhang, Bo Yuan, Baolin Bian, Haiyu Zhao, Anna Kiyomi, Hideki Hayashi, Yui Iwatani, Munetoshi Sugiura, Norio Takagi

**Affiliations:** ^1^Department of Applied Biochemistry, Tokyo University of Pharmacy & Life Sciences, Hachioji, Japan; ^2^Institute of Chinese Materia Medica, China Academy of Chinese Medical Sciences, Beijing, China; ^3^Laboratory of Pharmacology, School of Pharmacy, Faculty of Pharmaceutical Sciences, Josai University, Sakado, Japan; ^4^Department of Drug Safety and Risk Management, Tokyo University of Pharmacy & Life Sciences, Hachioji, Japan

**Keywords:** breast cancer cells, hellebrigenin, arenobufagin, apoptosis, necrosis, G_2_/M arrest, autophagy

## Abstract

Development of new therapeutic strategies for breast cancer is urgently needed due to the sustained emergence of drug resistance, tumor recurrence and metastasis. To gain a novel insight into therapeutic approaches to fight against breast cancer, the cytocidal effects of hellebrigenin (Helle) and arenobufagin (Areno) were investigated in human estrogen receptor (ER)-positive breast cancer cell line MCF-7 and triple-negative breast cancer cell line MDA-MB-231. Helle exhibited more potent cytotoxicity than Areno in both cancer cells, and MCF-7 cells were more susceptible to both drugs in comparison with MDA-MB-231 cells. Apoptotic-like morphological characteristics, along with the downregulation of the expression level of Bcl-2 and Bcl-xL and the upregulation of the expression level of Bad, were observed in Helle-treated MCF-7 cells. Helle also caused the activation of caspase-8, caspase-9, along with the cleavage of poly(ADP-ribose) polymerase in MCF-7 cells. Helle-mediated necrosis-like phenotype, as evidenced by the increased propidium iodide (PI)-positive cells was further observed. G_2_/M cell cycle arrest was also induced by Helle in the cells. Upregulation of the expression level of p21 and downregulation of the expression level of cyclin D1, cyclin E1, cdc25C and survivin were observed in MCF-7 cells treated with Helle and occurred in parallel with G_2_/M arrest. Autophagy was triggered in MCF-7 cells and the addition of wortmannin or 3-MA, two well-known autophagy inhibitors, slightly but significantly rescued the cells. Furthermore, similar alterations of some key molecules associated with the aforementioned biological phenomena were observed in MDA-MB-231 cells. Intriguingly, the numbers of PI-positive cells in Helle-treated MCF-7 cells were significantly reduced by wortmannin and 3-MA, respectively. In addition, Helle-triggered G_2_/M arrest was significantly corrected by wortmannin, suggesting autophagy induction contributed to Helle-induced cytotoxicity of breast cancer cells by modulating necrosis and cell cycle arrest. Collectively, our results suggested potential usefulness of both Helle and Areno in developing therapeutic strategies to treat patients with different types of breast cancer, especially ER-positive breast cancer.

## Introduction

According to estimates from the World Health Organization (WHO) in 2020, female breast cancer has surpassed other cancer types as the most commonly diagnosed cancer, with an estimated 2.3 million new cases (1). Estrogen receptor (ER), progesterone receptor (PR) and human epidermal growth factor receptor-2 (HER2) are the most important prognostic and predictive markers for determining the appropriate breast cancer treatment (2, 3). Despite recent advances in early detection, diagnosis, and targeted treatment options such as Herceptin (trastuzumab), breast cancer remains a major health problem and is still the leading cause of cancer death in women worldwide (1, 2). Thus, the development of new therapeutic strategies is urgently needed for the treatment of breast cancer.

Given inseparable relationship between cancer development and inflammation, many anticancer agents have been well characterized by their anti-inflammatory and anticancer activity (4–6). Cinobufacini (also known as Huachansu), a well-known traditional Chinese medicine that comes from the dried skin of *Bufo bufo gargarizans* Cantor, has long been successfully used in clinic as anti-inflammatory and anticancer agents in China (7–9). In line with previous reports, we recently demonstrated that indolealkylamines, a kind of important hydrophilic ingredients of cinobufacini, exhibited protective effect on LPS-induced inflammation in zebrafish (10, 11). Bufadienolides are another kind of important effective constituents of cinobufacini, and we also demonstrated that active bufadienolide compounds such as gamabufotalin, hellebrigenin (Helle) and arenobufagin (Areno) exhibited selective cytocidal effects against intractable cancer cells (12–14). Furthermore, we recently demonstrated that clinically achieved concentrations of trivalent arsenic derivative (As^III^) combined with gamabufotalin exhibited synergistic cytotoxicity against glioblastoma cell line U-87, whereas showed much less cytotoxicity to human normal peripheral blood mononuclear cells (PBMCs) (15). These findings thus provide fundamental insight into the mechanisms underlying the anti-inflammatory and anticancer activity of cinobufacini. Although Deng et al. have demonstrated that Areno inhibits the growth of a human breast cancer cell line MCF-7 by inducing apoptosis associated with JNK signaling pathway (16), the cytocidal effects of Helle and Areno against breast cancer cells as well as the underlying molecular mechanisms remain largely unexplored.

Apoptosis has been characterized by several morphologic features including cell shrinkage and chromatin condensation, all of which are linked to the activation of caspases and their downstream molecules such as poly(ADP-ribose) polymerase (PARP) (17, 18). The activation of caspase-8 and caspase-9 has been closely linked to two major apoptotic machinery, known as the extrinsic and intrinsic apoptotic pathway, respectively (17, 18). A series of Bcl-2 family members, including anti-apoptotic effectors such as Bcl-2/Bcl-xL, and pro-apoptotic effectors such as Bax/Bad, have been demonstrated to regulate apoptosis by modulating mitochondrial membrane permeabilization (18, 19). On the other hand, necrosis has also linked to anticancer activity of chemotherapeutic agents, and has received considerable attention for the treatment of apoptosis-resistant cancer cells, in which apoptotic pathway is suppressed or absent (20). Cell cycle arrest has been viewed as one of major underlying mechanisms for the cytotoxicity of various anticancer drugs. Cell cycle is known to be sophisticatedly controlled by cyclin-dependent kinases (CDK) paired with their respective cyclin binding partners (CDK/Cyclin complexes) (14, 21, 22). The alteration of p21 Waf1/Cip1 (p21) and p27 Kip1 (p27), known as inhibitors for CDK/Cyclin complexes, also closely links to cell cycle arrest (21–24). Cdc25C, a member of cdc25 family, is known to be associated with cell cycle transition by modulating cdc2/Cyclin B1 (14, 25). Moreover, survivin is highly expressed in most human cancer cells and implicated in cell cycle transitions (12, 14, 26). Besides, induction of autophagic cell death has emerged as a critical mechanism underlying cytocidal effect of various anticancer drugs (12, 14, 15). Although previous studies have demonstrated that the cytotoxicity of some bufadienolide compounds such as Helle and Areno are attributed to the induction of either of apoptosis/necrosis, cell cycle arrest and autophagy in hepatoma and glioblastoma cells (12, 27, 28), whether and how these biological phenomena contribute to the cytocidal effects of Helle and Areno in human breast cancer cells remain to be seen.

In this study, cytocidal effects of Helle and Areno were investigated in the human ER-positive breast cancer cell line MCF-7 and triple-negative breast cancer cell line MDA-MB-231, by focusing on growth inhibition associated with apoptosis/necrosis, cell cycle arrest and autophagic cell death. Key regulatory molecules involved in the above-mentioned biological phenomena were evaluated to further elucidate the underlying mechanisms. Wortmannin and 3-methyladenine (3-MA), two well-known autophagy inhibitors, were also employed to evaluate the correlation between autophagy and apoptosis/necrosis as well as cell cycle transition.

## Materials and Methods

### Materials

Hellebrigenin (Helle) (≥98% purity) and arenobufagin (Areno) (≥98% purity) were purchased from Baoji Herbest Bio-Tech Co., Ltd. (Baoji, China). Fetal bovine serum (FBS) and HEPES were purchased from Sigma Chemical Co. (St. Louis, MO, USA). RPMI-1640 and DMEM medium, 3-methyladenine (3-MA) and wortmannin were obtained from Wako Pure Chemical Industries (Osaka, Japan). 25% glutaraldehyde solution were purchased from Kanto chemical CO., INC. (Tokyo, Japan). WST-1 and 1-Methoxy PMS were obtained from Dojindo Molecular Technologies, Inc. (Tokyo, Japan). Propidium iodide (PI), ribonuclease A (RNaseA), crystal violet (C.I. 42555) Certistain^®^ were purchased from Merck KGaA (Sigma-Aldrich; Darmstadt, Germany).

### Cell Culture and Treatment

Human breast cancer cell line MCF-7 and MDA-MB-231 were obtained from the American Type Culture Collection (ATCC, Manassas, VA, USA). MCF-7 and MDA-MB-231 were cultured in RPMI-1640 and DMEM medium (high glucose) supplemented with 10% heat-inactivated FBS and antibiotics (100 U/ml of penicillin and 100 μg/ml of streptomycin (Wako Pure Chemical Industries)) in a humidified 5% CO_2_ atmosphere at 37°C, respectively. In experiments using inhibitors, MCF-7 and MDA-MB-231 cells were treated with respective inhibitor at the indicated concentrations for 30 min prior to treatment with indicated concentrations of Helle, in the presence or absence of respective inhibitor for an additional 48 h. Helle and Areno were dissolved in dimethyl sulfoxide (DMSO), and no cytotoxicity of the final concentrations of DMSO (0.02%) was observed in the current experimental system.

### Cell Viability and Clonogenic Survival

Following treatment for 48 h with various concentrations of Helle, cell viability was measured by the WST-1 assay as described previously (29). The relative cell viability was expressed as the ratio of the absorbance of each treatment group against those of the corresponding untreated control group. The IC_50_ values of the drugs were calculated using GraphPad Prism^®^7 software. With respect to the morphological alterations of U-87 cells, the cells were imaged using an inverted microscope (CKX53; Olympus Corporation, Tokyo, Japan) fitted with a digital camera following treatment with various concentrations (3, 10, 30 and 100 nM) of Helle for 48 h. Clonogenic survival assays were performed according to the methods previously described with slight modifications (12, 14, 24). Briefly, MCF-7 and MDA-MB-231 cells were seeded at 2,000 cells/well in 12-well plates, and treated for 24 h with various concentrations (1, 3, 10, 30, 100, 300 and 1000 nM) of Helle and Areno, respectively. The medium was then replaced with fresh media and the cells were allowed to grow for 7–10 days in a humidified 5% CO_2_ atmosphere at 37°C. Then, the cells were fixed with 0.25% glutaraldehyde/PBS for 15 min prior to staining with 0.2% crystal violet/PBS for 10 min at room temperature. Following washout of extra crystal violet with water to get an adequate staining pattern, the images of crystal violet-stained cells were scanned into a computer, followed by dissolution of the violet-stained cells in 1% SDS. The absorbance of the cell lysates was determined at 550 nm. The relative colony formation rate was expressed as the ratio of the absorbance at 550 nm of each treatment group against those of the corresponding untreated control group.

### Hoechst33342/PI Double Staining Assay

The phenotypic features of cell death were evaluated by use of Hoechst33342/PI double staining assay. After treatment for 48 h with different concentrations (3, 10, 30 and 100 nM) of Helle, MCF-7 cells were washed twice with cold PBS, followed by incubation with 100 μl of staining solution (3 μl/ml of PI and 0.05% Hoechst in PBS) for 15 min at room temperature. The staining images were captured using a BZ-X800 Keyence fluorescence microscope (Keyence, Osaka, Japan) and Leica X software at 100×magnification (Leica, Tokyo, Japan).

### Cell Cycle Analysis

After treatment of MCF-7 cells with various concentrations of Helle (10, 30 and 100 nM) for 48 h, cell cycle analysis was performed using a FACS Canto™ flow cytometer (Becton Dickinson, CA, USA) according to the methods reported previously (14, 15, 30). Briefly, cells were washed twice with cold PBS, fixed with 1% paraformaldehyde/PBS on ice for 30 min, washed twice again with cold PBS, permeabilized in 70% (v/v) cold ethanol and kept at -20°C for at least 4 h. The cell were then incubated with 0.25% Triton-X 100 for 5 min on ice. After centrifugation (430×g for 5 min at 4°C) and washing with PBS, cells were resuspended in 500 μl of PI/RNase A/PBS (5 μg/ml of PI and 0.1% RNase A in PBS) and incubated for 30 min in the dark at room temperature. A total of 10,000 events were acquired, and FACSDiva™ software (v6.0; BD Biosciences) and ModFit LT™ v3.0 (Verity Software House, Inc., Topsham, ME, USA) were used to calculate the number of cells at each G_0_/G_1_, S and G_2_/M phase fraction.

### Western Blot Analysis

For preparation of the protein samples, cell pellets (1-2×10^6^ cells per 110 μl buffer) were suspended in Laemmli buffer containing protease inhibitor cocktail tablets (Roche Diagnostics Co., Mannheim, Germany). Protein concentrations of the supernatant were determined according to Bradford’s method using the Protein Assay Dye Reagent Concentrate (Bio-Rad Laboratories, Inc.), according to the manufacturer’s instructions, using BSA as the standard. Western blot analysis was carried out according to the method previously described (14, 15). Protein bands were detected using the following primary antibodies: Mouse anti-human β-actin (cat. no. A-5441; Sigma-Aldrich; Merck KGaA, Darmstadt, Germany), mouse anti-human Bcl-2 (cat. no. 12741), rabbit anti-human Bcl-xL (cat. no. 2764), rabbit anti-human Bax (cat. no. 2772), rabbit anti-human Bad (cat. no. 9292), mouse anti-human caspase 8 (cat. no. 9746), rabbit anti-human caspase 9 (cat. no. 9502), rabbit anti-human PARP (cat. no. 9542), mouse anti-human p21 (cat. no. 2946), rabbit anti-human p27 (cat. no. 2552), rabbit anti-human cyclin B1 (cat. no. 4135), rabbit anti-human cyclin D1 (cat. no. 2978), rabbit anti-human cyclin E1 (cat. no. 20808), rabbit anti-human Cdc25c (cat. no. 4688), mouse anti-human survivin (cat. no. 2802), rabbit anti-human LC3A/B (cat. no. 12741), rabbit anti-human p-AMPK α (cat. no. 2537), rabbit anti-human AMPK α (cat. no. 2532; all from Cell Signaling Technology, Inc., Danvers, MA, USA)). Blotted protein bands were detected with respective horseradish peroxidase-conjugated secondary antibody (anti-mouse IgG, cat. no. A5906; anti-rabbit IgG, cat. no. A0545; both from Sigma-Aldrich; Merck KGaA) and an appropriate enhanced chemiluminescence (ECL) Western Blot detection kits [(ImmunoStar basic and ImmunoStar zeta (FUJIFILM Wako, Osaka, Japan) or West Femto (Pierce Biotechnology, Thermo Fisher, MA, USA)]. Relative amounts of the immunoreactive proteins obtained were subsequently quantitatively analyzed with the ImageJ software program (Rasband, W.S., ImageJ, U. S. National Institutes of Health, Bethesda, Maryland, USA, http://rsb.info.nih.gov/ij/).

### Statistical Analysis

Experiments were independently repeated three times, and the results were shown as the means ± standard deviation (SD) of three assays. The Student’s t-test was used to compare sample means from two groups, and one-way ANOVA followed by Dunnett’s *post hoc* test was used to compare sample means from more than three groups. A probability level of *p*<0.05 was considered to indicate a statistically significant difference.

## Results

### Cytotoxic Effects of Helle and Areno Against Human Breast Cancer Cell Lines MCF-7 and MDA-MB-231

**Figure 1:**
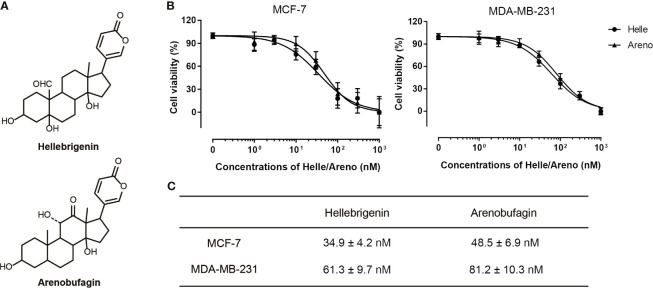
Cytotoxic effects of Helle and Areno against human breast cancer cell lines MCF-7 and MDA-MB-231. Chemical structures of Helle and Areno **(A)**. Cell viability was determined by WST-1 assay after treatment of MCF-7 or MDA-MB-231 **(B)** with various concentrations of Helle and Areno (1, 3, 10, 30, 100, 300 and 1000 nM) for 48 h. Relative cell viability was calculated as the ratio of the absorbance at 450 nm of each treatment group against those of the corresponding untreated control group. The IC_50_ values of the drugs were calculated using GraphPad Prism^®^7 software **(C)**. Data are shown as the means ± SD from more than three independent experiments.

A significant decrease in cell viability was observed in a dose-dependent manner in MCF-7 and MDA-MB-231 cells after treatment with various concentrations of Helle for 48 h, and its IC_50_ value was 34.9 ± 4.2 nM and 61.3 ± 9.7 nM, respectively (**Figures 1B, C**). A similar dose-dependent growth inhibition was also observed in both cells after treatment with various concentrations of Areno for 48 h, and its IC_50_ value was 48.5 ± 6.9 nM and 81.2 ± 10.3 nM, respectively (**Figures 1B, C**). These results indicated that the cytotoxicity of Helle was more potent than Areno, and that MCF-7 cells were more sensitive to the cytotoxicity of both Helle and Areno, compared to MDA-MB-231 cells.

### Inhibition of Colony Formation of MCF-7 and MDA-MB-231 Cells by Helle and Areno

**Figure 2:**
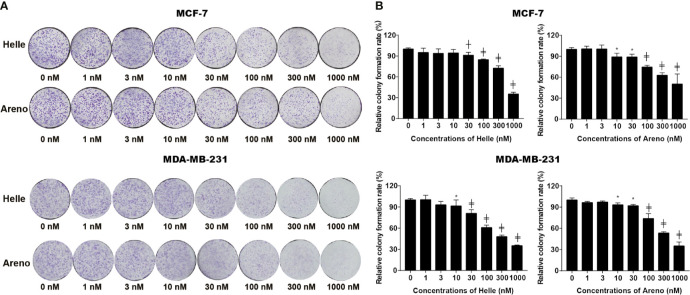
Inhibition of colony formation of MCF-7 and MDA-MB-231 cells by Helle and Areno. The cells were seeded at 2,000 cells/well in 12-well plates following treatment with indicated concentrations (1, 3, 10, 30, 100, 300 and 1000 nM) of Helle and Areno for 24 h. Representative images of the clonogenic assays are shown from three independent experiments **(A)**. The relative colony formation rate was expressed as the ratio of the absorbance at 550 nm of each treatment group against those of the corresponding untreated control group as described in Materials and methods **(B)**. Data are shown as means ± SD from three independent experiments. *p < 0.05; ^┼^p < 0.01; ^╪^p<0.0001 *vs.* control. Helle, hellebrigenin; Areno, arenobufagin.

A colony formation assay was conducted to evaluate whether exposure to Helle and Areno suppressed the surviving fraction of MCF-7 and MDA-MB-231 cells. As shown in **Figure 2**, significant suppression of the colony numbers of MCF-7 and MDA-MB-231 cells was observed after long-term treatment with Helle at concentrations starting from 30 nM and 10 nM, respectively. In comparison, an inhibitory activity against colony formation of both cells was observed in Areno even at the concentrations as low as 10 nM. These results indicated the superior potency of Helle and Areno against the survival of both MCF-7 and MDA-MB-231 cells.

### Phenotypic Features of Cell Death in MCF-7 Cells Treated by Helle

**Figure 3:**
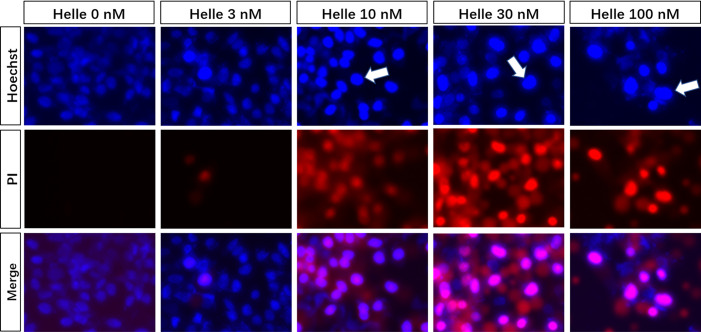
Phenotypic features of cell death in MCF-7 cells treated by Helle. After treatment with various concentrations of Helle (3, 10, 30 and 100 nM) for 48 h, the phenotypic features of cell death were evaluated using the Hoechst 33342 (blue)/PI (red) staining as described in Materials and methods. Cells with condensed nuclei (arrows) and red fluorescence were identified as those undergoing apoptosis and necrosis, respectively. The pink fluorescence represents the merged images of Hoechst 33342 and PI. Images were captured using a BZ-X800 Keyence fluorescence microscope and Leica X software at 100 × magnification. Helle, hellebrigenin.

Due to the potent cytotoxic effect of Helle and higher susceptibility of MCF-7 cells to the drug (**Figure 1**), the mechanism underlying its cytotoxicity was thus further evaluated in the cells. Following treatment for 48 h with various concentrations of Helle (3, 10, 30 and 100 nM), which were determined according to its IC_50_ value, the phenotypic features of cell death were evaluated by use of Hoechst33342/PI staining assay. As shown in **Figure 3** and **Supplementary Figure 1**, following exposure to 10 nM Helle, a non-negligible number of MCF-7 cells showing exclusively apoptotic-like morphology, characterized by cell shrinkage, chromatin condensation as evidenced by a clear increase in the fluorescence intensity of Hoechst33342 in comparison with control group, were observed, although a clear alteration was not recognized after treatment with 3 nM Helle. Intriguingly, a small portion of PI-positive cells were observed after treatment with Helle even at the concentrations as low as 3 nM, indicating that Helle-triggered cytotoxicity was also associated with a necrosis-like phenotype in the cells. Furthermore, these phenomena associated with apoptosis and/or necrosis-like phenotype became more evident when the concentrations were greater than 30 nM.

### Helle-Mediated Activation of Apoptosis Signaling Pathway in MCF-7 Cells

**Figure 4:**
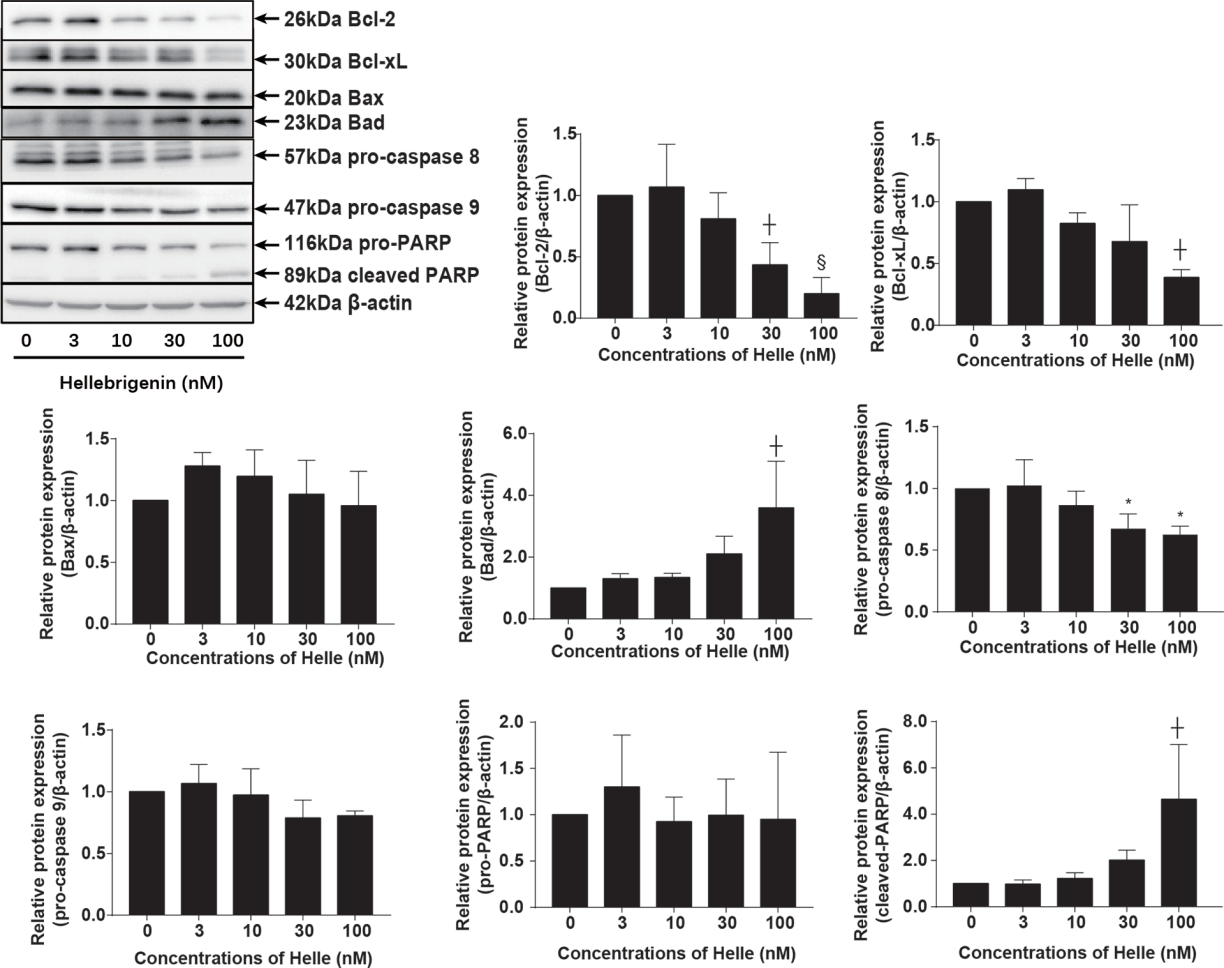
Helle-mediated activation of apoptosis signaling pathway in MCF-7 cells. After treatment with various concentrations of Helle (3, 10, 30 and 100 nM) for 48 h, the expression profile of apoptosis−related proteins was analyzed using western blotting. The relative expression levels were expressed as the ratios between each target gene protein and β-actin protein expression levels, and compared with those of untreated control group, respectively. Data are presented as the means ± SD from three independent experiments. *p < 0.05; ^┼^p < 0.01; ^§^p < 0.001 *vs.* control.

Commitment of cells to apoptosis is closely regulated by Bcl-2 (B-cell leukemia/lymphoma) family, including anti-apoptotic effectors such as Bcl-2/Bcl-xL, and pro-apoptotic effectors such as Bax/Bad (19). As shown in **Figure 4**, in comparison to control group, a dose-dependent downregulation of the expression level of Bcl-2 and Bcl-xL was induced by Helle. Coincidentally, a dose-dependent upregulation of the expression level of Bad was observed in Helle-treated MCF-7 cells, although almost no significant alteration in the expression level of Bax was detected. At the same time, the exposure to Helle caused a significant downregulation of the expression of pro-caspase-8, which was accompanied by a trend towards downregulation of the expression of pro-caspase-9, indicating the activation of caspase-8 and caspase-9. Furthermore, cleavage of PARP, known as an early marker of chemotherapy-induced apoptosis (31), was observed concomitantly, indicating the onset of apoptosis. In addition, similar downregulation of the expression levels of Bcl-2, Bcl-xL, as well as pro-caspase-8 and -9 was also observed in MDA-MB-231 cells treated by Helle (**Supplementary Figure 2**).

### Effect of Helle on the Cell Cycle Profiling and the Expression Level of Cell Cycle Related-Proteins in MCF-7 Cells

**Figure 5:**
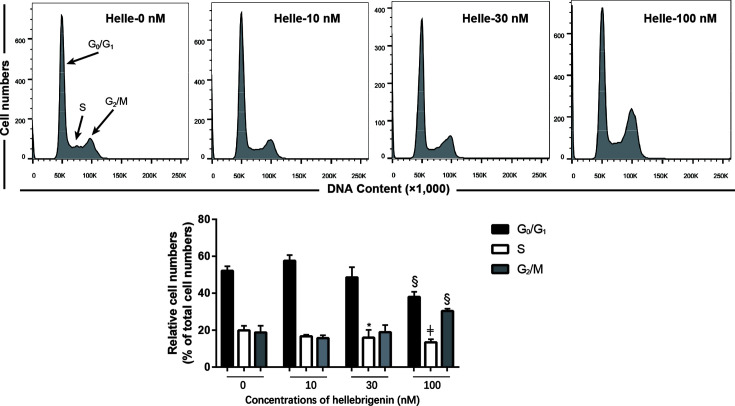
Effect of Helle on the cell cycle profiling in MCF-7 cells. After treatment with various concentrations of Helle (10, 30 and 100 nM) for 48 h, cell cycle analysis was performed using a FACS Canto flow cytometer as described in Materials and methods. A representative FACS histogram from three independent experiments is shown. ModFit LT™ v3.0 was used to calculate the number of cells at each G_0_/G_1_, S and G_2_/M phase fraction. Results are shown as the means ± SD from three independent experiments. *p < 0.05; ^§^p < 0.001; ^╪^p < 0.0001 *vs.* control. Helle, hellebrigenin.

To investigate whether cell cycle arrest is implicated in the cytocidal effect of Helle, cell cycle analyses were carried out using flow cytometry following treatment with various concentrations of Helle for 48 h. As shown in **Figure 5**, in comparison to control group, a modest increase in the number of cells in the G_2_/M phase along with a significant decrease in the number of cells in the S phase was observed in MCF-7 cells treated with Helle at the concentration starting from 30 nM. An increase in the number of cells in the G_2_/M phase was further strengthened following exposure to 100 nM Helle. Concomitantly, a significant decrease in the number of cells in G_0_/G_1_ and S phase was also observed. In addition, similar G_2_/M arrest was also observed in MDA-MB-231 cells following treatment for 48 h with 60 nM Helle, which was almost equal to its IC_50_ value of the cells (**Supplementary Figure 3**).

**Figure 6:**
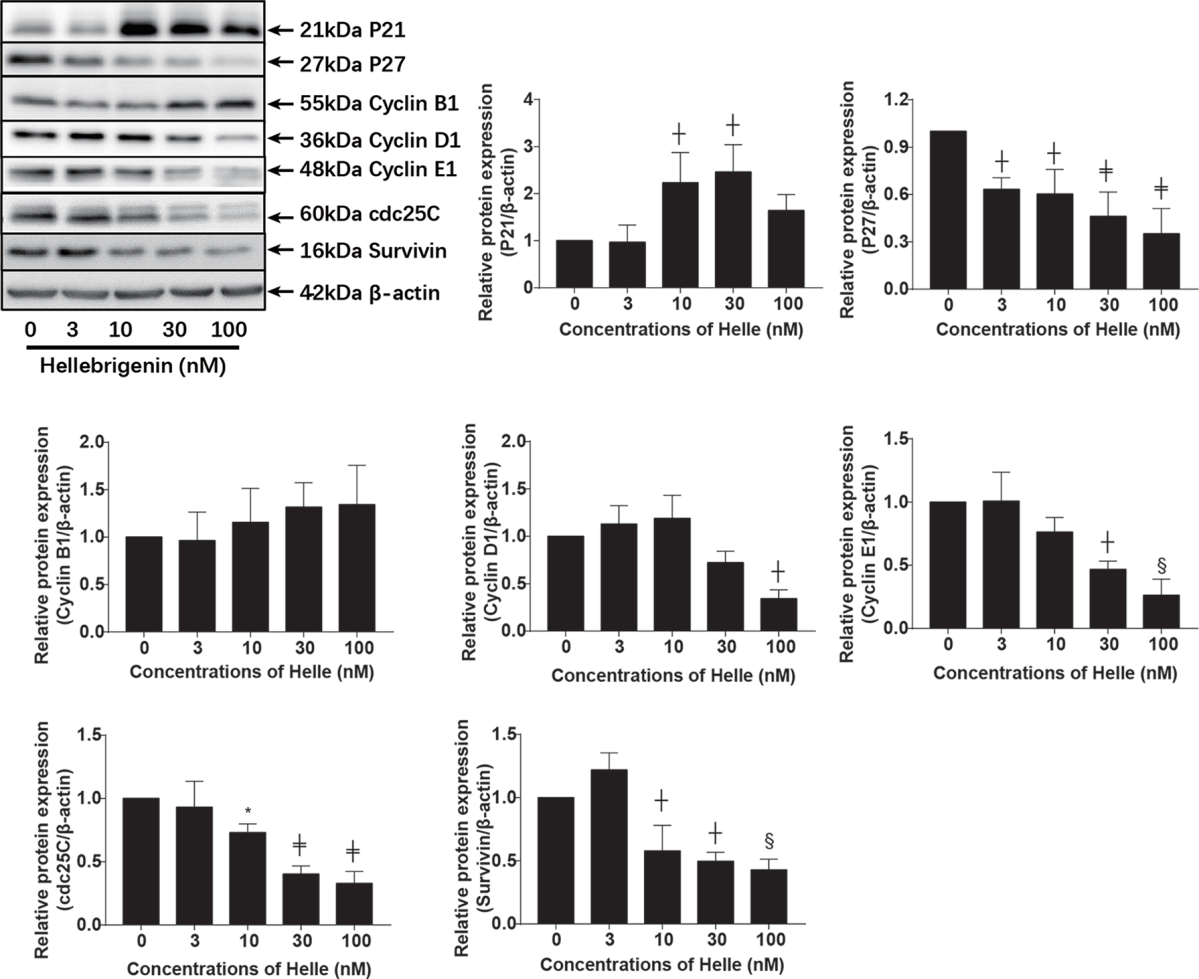
Effect of Helle on the expression level of cell cycle related-proteins in MCF-7 cells. After treatment with various concentrations of Helle (3, 10, 30 and 100 nM) for 48 h, the expression levels of cell cycle-related proteins were analyzed by western blotting. The relative expression levels were expressed as the ratios between each target gene protein and β-actin protein expression levels, and compared with those of untreated control group, respectively. Data are presented as the means ± SD from three independent experiments. *p < 0.05; ^┼^p < 0.01; ^§^p < 0.001; ^╪^p < 0.0001 *vs.* control.

Upregulation of p21 and p27 has been demonstrated to be involved in G_2_/M arrest induced by anticancer agents in different types of cancer cells including breast cancer (23, 24, 30). As shown in **Figure 6**, in line with these previous reports, a significant increase in the expression level of p21 was observed in MCF-7 cells treated with Helle at the concentration starting from 10 nM, although the magnitude of increase was different according to different drug doses. Surprisingly, downregulation of the expression level of p27 was observed in the treated cells, indicating its little involvement in Helle-mediated G_2_/M arrest. In addition, downregulation of the expression level of cyclin D1, cyclin E1 and cdc25C was observed in Helle-treated MCF-7 cells in a dose-dependent manner, whereas almost no alteration of cyclin B1 expression was detected. Of note, the exposure to Helle at the concentrations greater than 10 nM potently downregulated the expression level of survivin. Furthermore, similar downregulation of the expression level of cyclin D1, cyclin E1 as well as cdc25C was observed in Helle-treated MDA-MB-231 cells (**Supplementary Figure 4**).

### Involvement of Autophagic Cell Death in Helle-Triggered Cytotoxicity in MCF-7 Cells

**Figure 7:**
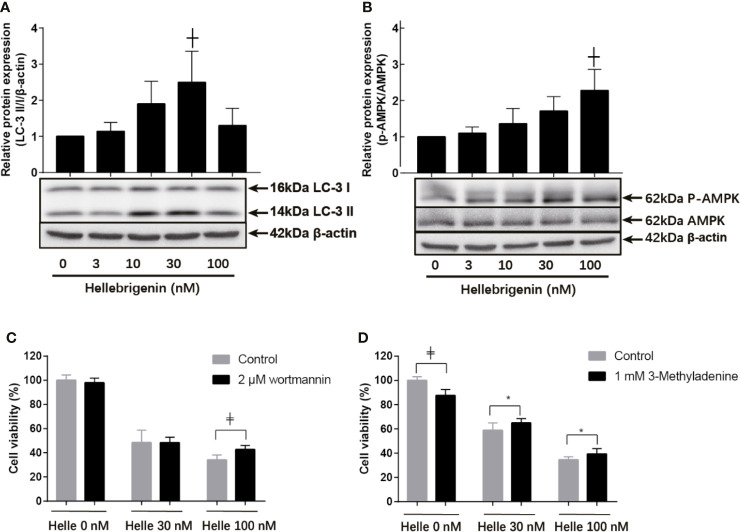
Involvement of autophagic cell death in the cytotoxicity of MCF-7 cells treated with Helle. After treatment with various concentrations of Helle (3, 10, 30 and 100 nM) for 48 h, the expression levels of autophagy induction−related proteins were analyzed by western blotting **(A, B)**. Cell viability was determined by WST-1 assay after treatment for 48 h with Helle at the concentrations of 30 or 100 nM in the absence or presence of 2 μM wortmannin **(C)** and 1 mM 3-MA **(D)**, respectively. Data are presented as the means ± SD from three independent experiments. *p < 0.05; ^┼^p < 0.01; ^╪^p < 0.0001 *vs.* control. Helle, hellebrigenin.

Many anticancer agents have been characterized as inducers of autophagy (14, 24, 30), and LC3 has been well known as an established autophagy marker (32, 33). As shown in **Figure 7A**, a modest upregulation of the expression level of LC3 was clearly induced by the treatment with 10 nM Helle compared to control group. Furthermore, exposure to 30 nM Helle significantly upregulated the expression level of LC3. Surprisingly, the magnitude expression level of LC3 dropped when the concentrations of Helle increased up to 100 nM. The drop might be due to the degradation of LC3, as a result of intensive cytotoxicity of 100 nM Helle, although further investigation is obviously needed. Additionally, a similar upregulation of the expression level of LC3 was also observed in MDA-MB-231 cells treated by Helle (**Supplementary Figure 5**). AMP-activated protein kinase (AMPK), a key energy sensor, has been shown to be an upstream promoter of autophagy induction (32, 34). In this regard, a dose-dependent increase in the expression level of phospho-AMPK over the endogenous level was detected in the treated cells, and a statistically significant increase in its expression was further observed in 100 nM Helle-treated cells (**Figure 7B**). Moreover, almost no change in the expression level of total AMPK expression was observed (**Figure 7B**).

Next, two well-known autophagy inhibitor, wortmannin and 3-methyladenine (3-MA) were employed to evaluated whether the induction of autophagy contributed to Helle-mediated cell growth inhibition. Consistent with **Figure 1**, Helle-triggered dose-dependent growth inhibition was reconfirmed in MCF-7 cells (**Figures 7C, D**). In comparison with control group, the addition of wortmannin significantly abrogated the cytotoxicity of 100 nM Helle, although similar abrogation was not observed when treated with 30 nM Helle (**Figure 7C**). Moreover, the addition of 3-MA rescued the cell from Helle-triggered toxicity, as evidenced by a modest but statistically significant increase in cell viability in the presence of 3-MA, although a slight growth inhibition was induced by 3-MA itself (**Figure 7D**).

### Correlation Between Autophagy and Apoptosis, Necrosis as Well as Cell Cycle Arrest in MCF-7 Cells Treated With Helle

**Figure 8:**
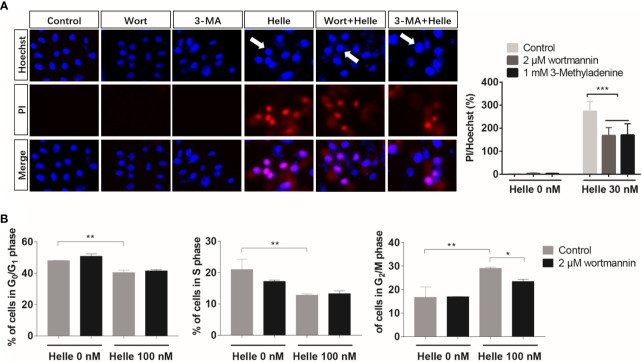
Correlation between autophagy and apoptosis, necrosis as well as cell cycle arrest in MCF-7 cells treated with Helle. **(A)** After treatment for 48 h with 30 nM Helle in the absence or presence of 2 μM wortmannin or 1 mM 3-MA, the phenotypic features of cell death were evaluated using the Hoechst 33342 (blue)/PI (red) staining as described in Materials and methods. The pink fluorescence represents the merged images of Hoechst 33342 and PI. Images were captured using a BZ-X800 Keyence fluorescence microscope and Leica X software at 100×magnification. **(B)** After treatment for 48 h with 100 nM Helle in the absence or presence of 2 μM wortmannin, cell cycle analysis was performed by the same manner as described in the legend of **Figure 5**. Data are presented as the means ± SD from three independent experiments. *p < 0.05; **p < 0.01; ***p < 0.001. Helle, hellebrigenin; Wort, wortmannin.

Therapeutic effects of anticancer drugs have been attributed to the crosstalk between apoptosis, necrosis and autophagy (17, 35). Wortmannin and 3-MA were thus employed to clarify whether there was a link between autophagy and apoptosis/necrosis as well as G_2_/M arrest in Helle-treated MCF-7. Consistent with **Figure 3**, apoptosis and necrosis, as evidenced by chromatin condensation and/or nuclei fragmentation, and the existence of PI-positive cells, were reconfirmed respectively in MCF-7 cells following the exposure to 30 nM Helle for 48 h (**Figure 8A**). In comparison, the numbers of PI-positive cells were significantly reduced by the addition of either wortmannin or 3-MA. On the other hand, almost no alteration was observed in the morphological changes associated with apoptosis regardless of the presence of wortmannin or 3-MA, indicating little relation between autophagy and apoptosis in Helle-treated MCF-7 cells. With respect to cell cycle arrest, G_2_/M phase arrest was confirmed in MCF-7 cells treated by 100 nM Helle, accompanied by a significant decrease in the number of cells in G_0_/G_1_ and S phases (**Figure 8B** and **Supplementary Figure 6**). Again, Helle-triggered G_2_/M-phase arrest was also successfully corrected by the addition of wortmannin.

## Discussion

Results from this study clearly demonstrated the cytotoxicity of Helle and Areno against human breast cancer cells, and further clarified that MCF-7 cells were more sensitive to the cytotoxicity of both compounds, compared to MDA-MB-231 cells. We have previously demonstrated that both Helle and Areno exhibit selective cytocidal effects against intractable cancer cells such as glioblastoma cell line U-87 and pancreatic cancer cell line SW1990, rather than noncancerous cells including human normal PBMCs (12–14), suggesting their broad-spectrum utility across different types of cancer cells. Given a clear difference between MCF-7 and MDA-MB-231 cells in terms of the expression level of ER (24, 30), our results further suggested potential usefulness of both Helle and Areno in developing therapeutic strategies to treat patients with different types of breast cancer, especially ER-positive breast cancer.

It has been demonstrated that high expression of steroid receptor coactivator 3 (SRC-3), known to play a critical role in mammary tumor development and metastasis (36, 37), is correlated with poor survival in ER-positive breast cancer patients (38, 39). A previous study has clarified that bufalin, one of active bufadienolide compounds with similar chemical structure with Helle and Areno, can function as a SRC-3 inhibitor by directly binding to SRC-3 in its receptor interacting domain and selectively promoting SRC-3 protein degradation in ER-positive breast cancer cell lines (40). Intriguingly, both bufalin and Areno have been demonstrated to suppress the proliferation and survival of HER2 overexpressing breast cancer cells, along with the declination of SRC-3 (41), although the effect of Helle on the expression level of SRC-3 still remains unknown. Taking these previous results and our observations into account, we thus suggest that the differential sensitivity of MCF-7 and MDA-MB-231 to both Helle and Areno could be attributed to the inhibitive effect of both two compounds on SRC-3, although the alteration of the expression and activity of SRC-3 in breast cancer *in vitro* and *in vivo* obviously warrants further investigation to draw a solid conclusion.

The induction of apoptosis and/or necrosis in cancer cells has been closely linked to the cytocidal effect of anticancer reagents including bufadienolide compounds such as Helle and Areno (12–14, 16, 27). In line with these previous reports, apoptotic-like morphological characteristics, along with the downregulation of Bcl-2 and Bcl-xL expression and the upregulation of Bad expression, were observed in MCF-7 cells treated with Helle. Similar alterations were also observed in MDA-MB-231 cells. The activation of caspase-8 and -9 as well as their downstream molecule, PARP, was further confirmed. Caspase-8 and -9 have been established as key regulators of intrinsic and extrinsic apoptosis pathway, respectively (18, 22). Collectively, we suggested that apoptosis induction *via* the activation of intrinsic/extrinsic apoptotic signaling pathway contributed to Helle-triggered cytotoxicity of breast cancer cells. Additionally, in agreement with our previous reports showing the necrosis-inducing activities of Helle against glioblastoma and pancreatic cancer cell lines (12, 13), induction of necrosis was also observed in MCF-7 cells. Given that tumor cells can evolve diverse strategies to evade apoptosis during tumor development (20, 42), the potential necrosis-inducing activities of Helle should be beneficial to treat cancer cells harboring the innate and/or adaptive resistance to apoptosis induced by anticancer reagents.

We further demonstrated that Helle-mediated G_2_/M arrest was observed in not only MCF-7 but also MDA-MB-231 cells. Similarly, G_2_/M arrest of hepatocellular carcinoma HepG2 cells and glioblastoma U-87 cells was also induced by Helle (12, 27), suggesting the generality of the mechanism underlying the cytotoxicity of Helle against different types of cancer cells. Furthermore, upregulation of p21 along with the downregulation of cyclin D1, cyclin E1 and cdc25C was observed in Helle-treated MCF-7 cells. Similar alterations of some aforementioned key molecules in the regulation of cell cycle were also confirmed in Helle-treated MDA-MB-231 cells. Upregulation of p21, a central player in the regulation of cell cycle, has been involved in the G_2_/M arrest of various cancer cells induced by diverse anticancer agents including active bufadienolide compounds (12, 14, 22, 24, 43). In addition, previous reports have demonstrated that downregulation of cdc25C occurred in parallel with G_2_/M arrest of hepatoma and glioblastoma cells induced by Helle and Areno (12, 27, 44). Downregulation of cyclin D1 and cyclin E1 has also been involved in the G_2_/M arrest of prostate cancer cell lines C4-2B and DU145 induced by resveratrol combined with docetaxel (23). Moreover, an ethanol extract of a traditional Chinese medicine, *Eupolyphaga sinensis* Walker induced G_2_/M arrest of a chronic myeloid leukemia cell line K562 accompanying through downregulation of cyclin D1, cyclin E1 and cdc25C (21). Besides, the fact that the suppression of survivin expression by Helle in MCF-7 cells was in good agreement with our previous report, in which Helle induced significant downregulation of survivin along with G_2_/M arrest of U-87 cells (12). Of note, Li et al. have also demonstrated that silencing of survivin expression causes reduced proliferation and G_2_/M cell cycle arrest in human cancer cells, including HeLa and MCF-7 cells (26). Collectively, our results suggested that Helle triggered G_2_/M arrest by modulating aforementioned key players in mitotic progression, and consequently resulted in growth inhibition of cancer cells.

A growing body of evidence has linked autophagic cell death to therapeutic efficacy of various anticancer drugs, and LC3 has been used ubiquitously as autophagy marker (14, 24, 30, 32, 33, 45). In agreement with these previous reports, we demonstrated the activation of AMPK, an upstream promoter of autophagy induction, and the upregulation of LC3 expression in MCF-7 cells treated with Helle. Our results also showed similar upregulation of LC3 expression in Helle-treated MDA-MB-231 cells. We further clarified that the addition of wortmannin and 3-MA slightly but significantly rescued MCF-7 cells. Intriguingly, natural products, which harbor anticancer properties related to their autophagy-inducing activity, have been recently demonstrated to sensitize breast cancer cells such as MDA-MB-231 cells to Taxol by inducing autophagic cell death (46, 47). Magnoflorine, a quaternary alkaloid isolated from Chinese herb, has also been demonstrated to improve the sensitivity of both MCF-7 and MDA-MB-231 cells to doxorubicin *via* inducing apoptosis and autophagy (48). Our results thus suggested that Helle could serve as a promising candidate of potent inducer of apoptosis and/or autophagic cell death, sensitizing breast cancer cells to conventional anticancer drugs such as Taxol and doxorubicin, although further studies will be needed to clarify the molecular details of Helle-triggered cytotoxicity in both cells.

The crosstalk between autophagy, necrosis and cell cycle arrest has received increasing attention to develop new therapeutic approaches for treatment of cancer patients (17, 35, 49, 50). In this regard, we demonstrated a close relation between autophagy and necrosis, as evidenced by a significant reduction of the numbers of PI-positive cells in MCF-7 cells when treated with Helle in the presence of wortmannin or 3-MA. We also demonstrated that Helle-triggered G_2_/M arrest was significantly corrected by wortmannin. Taking the previous findings and our results into account, we suggested that autophagy induction contributed to Helle-induced cytotoxicity of breast cancer cells by modulating necrosis induction and cell cycle arrest. Of note, we recently clarified that autophagy induction linked to S-phase arrest, rather than apoptosis and necrosis, in MDA-MB-231 cells treated with the combination of arsenite and tetrandrine, a Chinese plant-derived alkaloid (30, 51). Collectively, whether a correlation exists between autophagy, apoptosis/necrosis and cell cycle arrest seems to be highly dependent on different types of cancer cells and external stimuli.

## Conclusion

Our results suggest that the generality of the mechanism underlying the cytotoxicity of Helle against both breast cancer cells is linked to its apoptosis-, G_2_/M arrest- and autophagy-inducing activity. We further suggest potential usefulness of both Helle and Areno, two active bufadienolide compounds, in developing therapeutic strategies to fight against different types of breast cancer, especially ER-positive breast cancer. In addition to apoptosis induction, autophagy appeared to contribute to the cytotoxicity of Helle by modulating both necrosis and cell cycle arrest. Combined treatment has been widely used for cancer chemotherapy, aiming to maximize efficacy of anticancer drugs and minimize their undesirable side effects. In fact, Dong et al. have demonstrated that bufadienolide compounds such as gamabufotalin and bufalin sensitize human breast cancer cells to TRAIL-induced apoptosis (52). We also recently suggested that gamabufotalin could serve as a promising adjuvant therapeutic agent to potentiate therapeutic effect of arsenite in glioblastoma cells (15). The studies on cytocidal effects of conventional anticancer drugs in combination of Helle or Areno are ongoing in our laboratory.

## Data Availability Statement

The original contributions presented in the study are included in the article/**Supplementary Material**. Further inquiries can be directed to the corresponding authors.

## Author Contributions

BY and YZ designed the study and drafted the manuscript. YZ performed the experiments. BB, HZ, AK, YI, and MS assisted interpretation of the results. HH and NT contributed analytical tools and discussed the results. All authors contributed to the article and approved the submitted version.

## Funding

This work was partially supported by The Japan Society for the Promotion of Science (JSPS) KAKENHI Grant to BY (Grant Numbers 26460233) (Grant Numbers 17K08465). This study was also supported in part by grants from China Scholarship Council (file no. 201908110322).

## Conflict of Interest

The authors declare that the research was conducted in the absence of any commercial or financial relationships that could be construed as a potential conflict of interest.

## Publisher’s Note

All claims expressed in this article are solely those of the authors and do not necessarily represent those of their affiliated organizations, or those of the publisher, the editors and the reviewers. Any product that may be evaluated in this article, or claim that may be made by its manufacturer, is not guaranteed or endorsed by the publisher.
