# Supplementary material for: Cytotoxic Effects of Hellebrigenin and Arenobufagin Against Human Breast Cancer Cells
Source: Front Oncol. 2021 Aug 26;11:711220. doi: 10.3389/fonc.2021.711220 (PMC8427765; doi:10.3389/fonc.2021.711220)
Supplement: Supplementary file 1 [file DataSheet_1.docx]

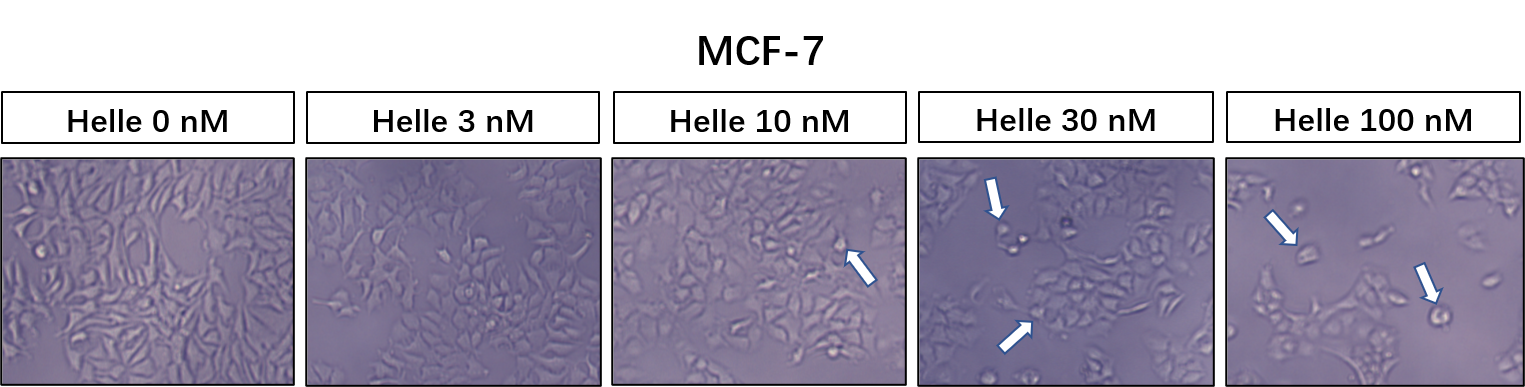


**Supplementary Figure 1.** Morphological alterations of Helle-treated MCF-7 cells. After treatment with various concentrations of Helle (3, 10, 30 and 100 nM) for 48 h, the morphological alterations of MCF-7 were evaluated as described in Materials and methods. The arrows show the shrinking cells following the treatment with Helle. Representative images of the morphological alterations are shown from three independent experiments. Images were captured using an inverted microscope (CKX53; Olympus Corporation, Tokyo, Japan) at 100 × magnification. Helle, hellebrigenin.


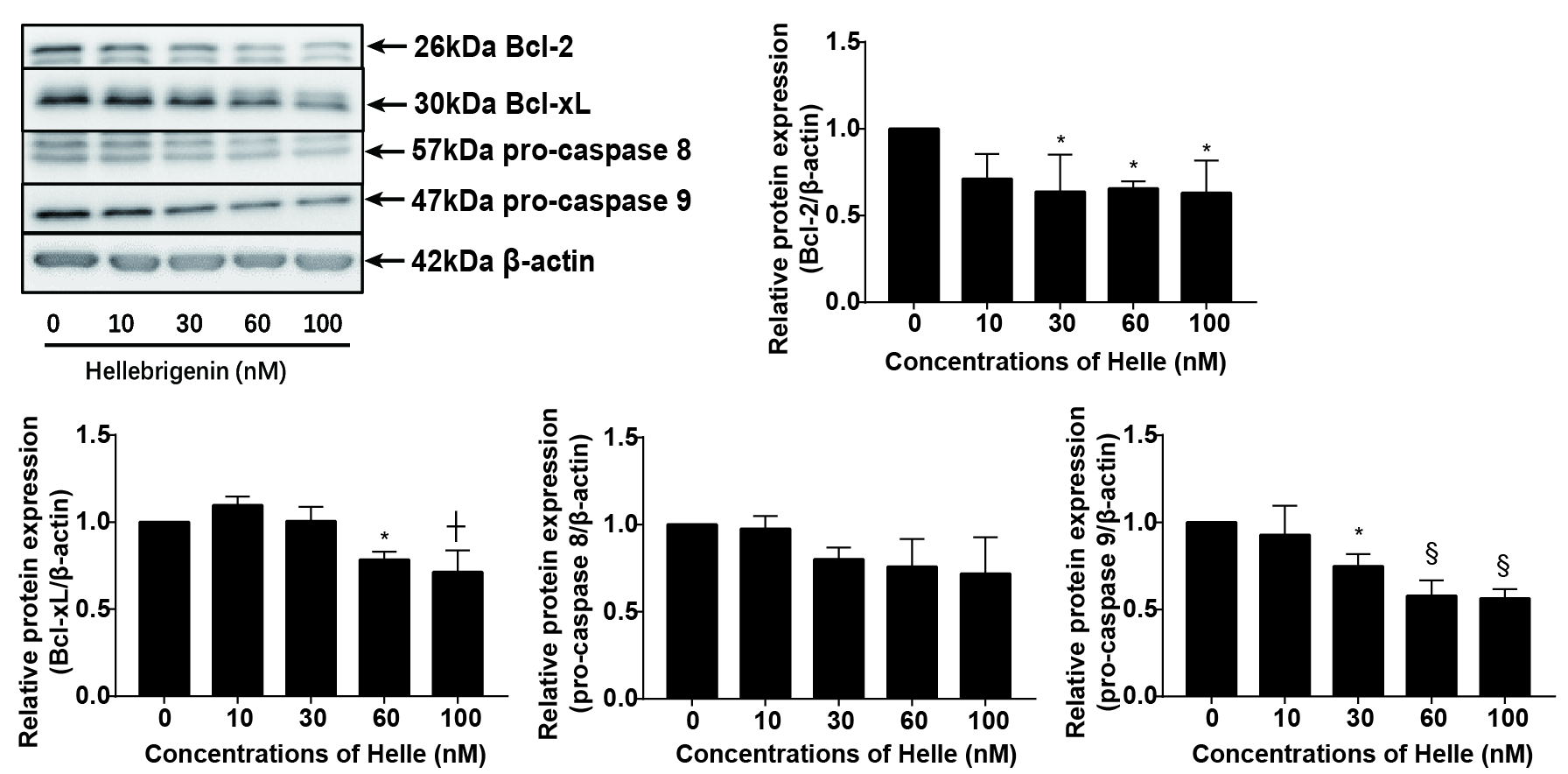


**Supplementary Figure 2.** Helle-mediated activation of apoptosis signaling pathway in MDA-MB-231 cells. After treatment with various concentrations of Helle (10, 30, 60, and 100 nM) for 48 h, the expression profile of apoptosis‑related proteins was analyzed using western blotting. The relative expression levels were expressed as the ratios between each target gene protein and β-actin protein expression levels, and compared with those of untreated control group, respectively. Data are presented as the means ± SD from three independent experiments. *, p<0.05; ┼, p<0.01; §, p<0.001 vs. control. Helle, hellebrigenin.


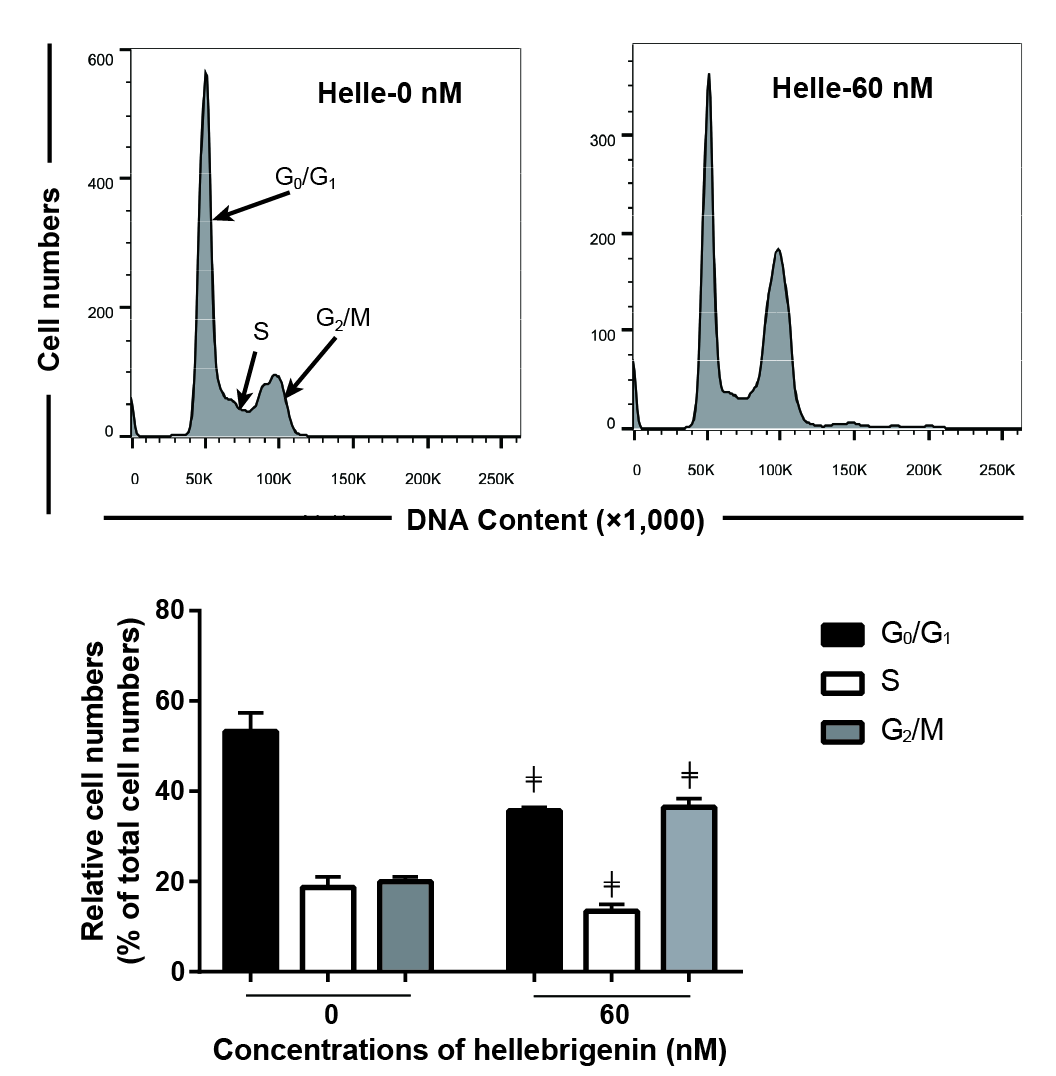


**Supplementary Figure 3.** Effect of Helle on the cell cycle profiling in MDA-MB-231 cells. After treatment for 48 h with 60 nM Helle, which was almost equal to its IC_50_ value of the cells, cell cycle analysis was performed using a FACS Canto flow cytometer as described in Materials and methods. A representative FACS histogram from three independent experiments is shown. ModFit LT™ v3.0 was used to calculate the number of cells at each G_0_/G_1_, S and G_2_/M phase fraction. Results are shown as the means ± SD from three independent experiments. ╪, p<0.0001 vs. control. Helle, hellebrigenin.


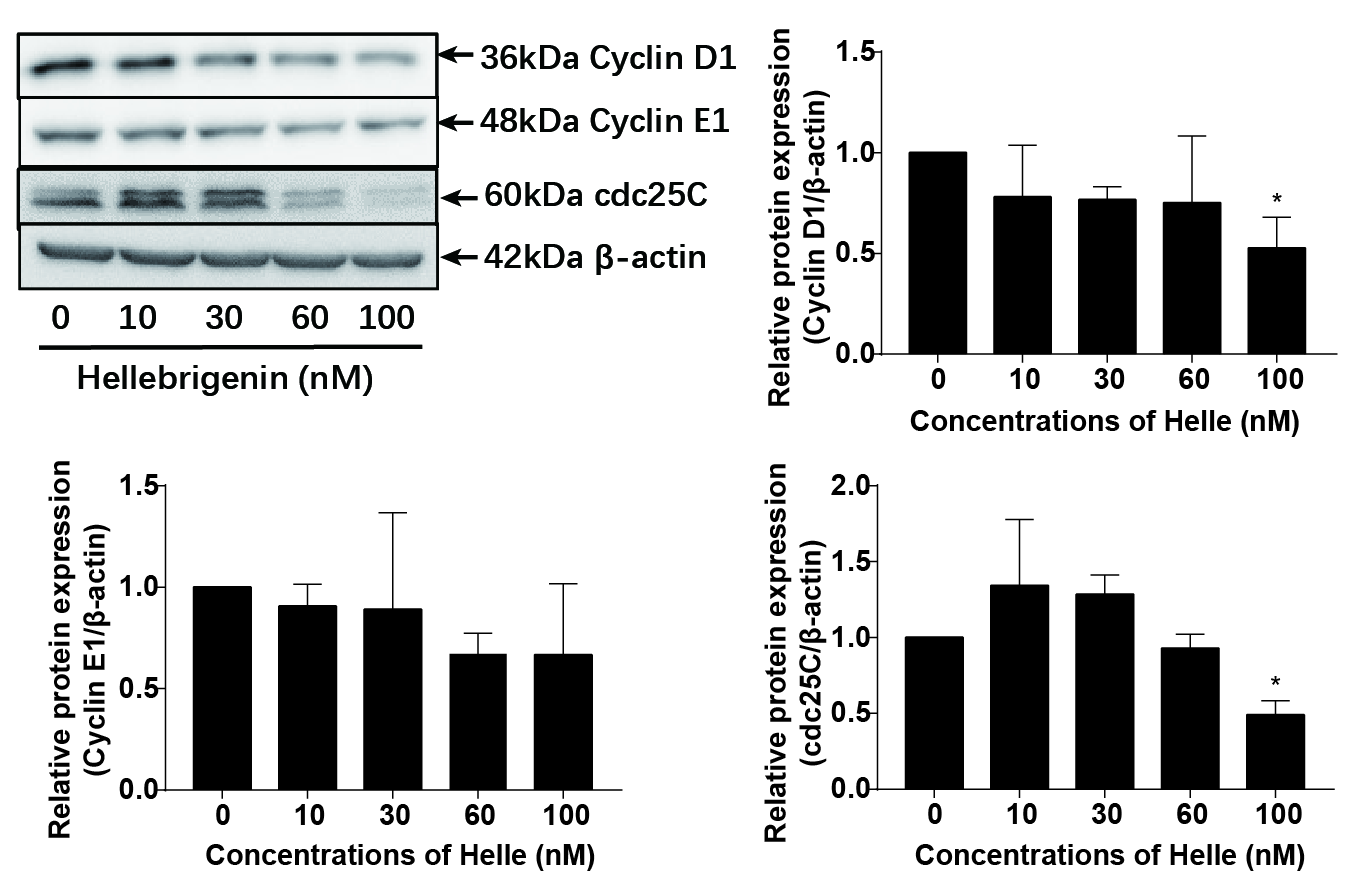


**Supplementary Figure 4.** Effect of Helle on the expression level of cell cycle related-proteins in MDA-MB-231 cells. After treatment with various concentrations of Helle (10, 30, 60 and 100 nM) for 48 h, the expression levels of cell cycle-related proteins were analyzed by western blotting. The relative expression levels were expressed as the ratios between each target gene protein and β-actin protein expression levels, and compared with those of untreated control group, respectively. Data are presented as the means ± SD from three independent experiments. *, p<0.05 vs. control. Helle, hellebrigenin.


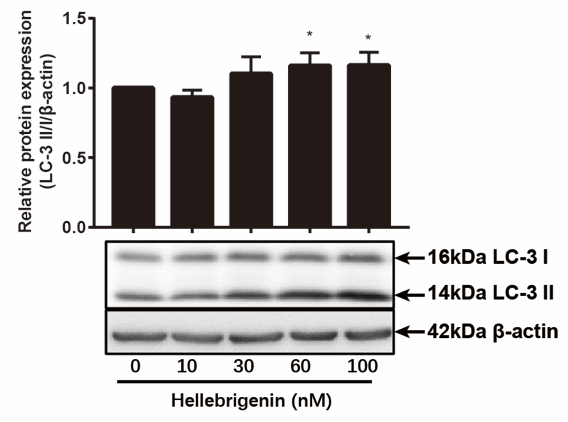


**Supplementary Figure 5.** Upregulation of the expression level of LC3 in MDA-MB-231cells treated with Helle. After treatment with various concentrations of Helle (10, 30, 60 and 100 nM) for 48 h, the expression level of LC3, an autophagic marker, was analyzed by western blotting. The relative expression level was expressed as the ratio between LC3 protein and β-actin protein expression levels, and compared with that of untreated control group, respectively. Data are presented as the means ± SD from three independent experiments. *, p<0.05 vs. control.


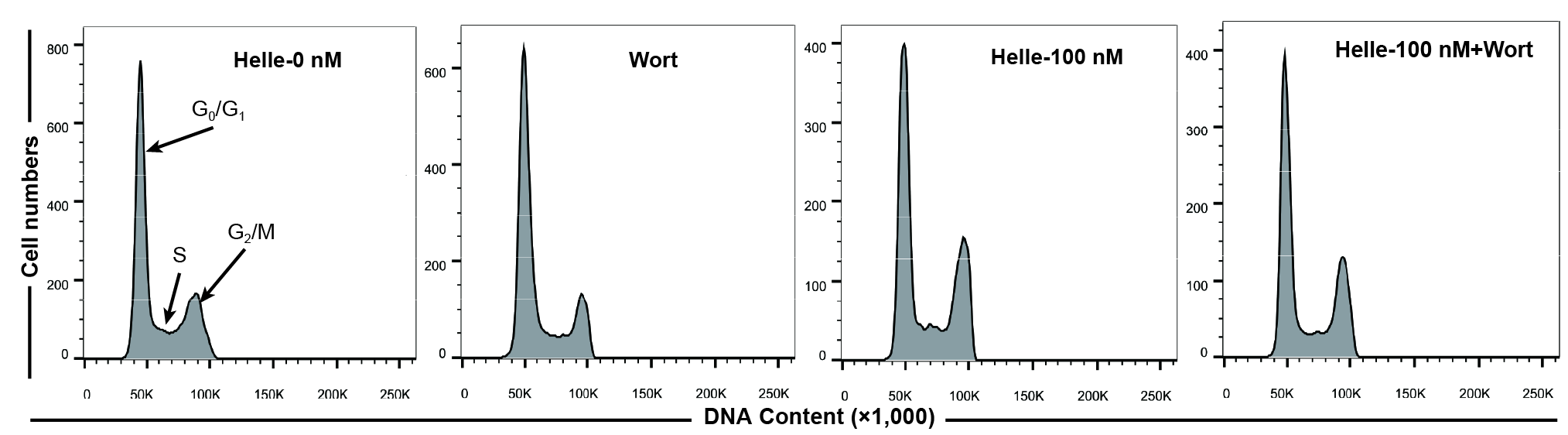


**Supplementary Figure 6.** Cell cycle profiling of MCF-7 cells treated by Helle with or without wortmannin. After treatment for 48 h with 100 nM Helle in the absence or presence of 2 μM wortmannin, cell cycle analysis was performed by the same manner as described in Materials and methods. A representative FACS histogram from three independent experiments is shown. Helle, hellebrigenin; Wort, wortmannin.
